# Implementation determinants of HIV Self-Testing among young sexual minority men

**DOI:** 10.1186/s13690-023-01126-y

**Published:** 2023-06-21

**Authors:** Juan Pablo Zapata, Andrew E. Petroll, Katherine G. Quinn, Alithia Zamantakis, Steven A. John

**Affiliations:** 1 Institute for Sexual and Gender Minority Health and Wellbeing, Feinberg School of Medicine, Northwestern University, 625 N Michigan Ave 14th Floor, Chicago, IL 60611 USA; 2 Health Intervention Sciences Group, Center for AIDS Intervention Research, Department of Psychiatry and Behavioral Medicine, Medical College of Wisconsin, Milwaukee, WI USA; 3 Division of Infectious Diseases, Department of Medicine, Medical College of Wisconsin, Milwaukee, WI USA

**Keywords:** HIV, HIV self-testing, HIVST, Sexual minority men, Men who have sex with men, Determinants, CFIR

## Abstract

**Background:**

HIV self-testing (HIVST) has shown the potential for reaching people with heightened vulnerability to HIV, including young sexual minority men (YSMM), yet implementation of HIVST among YSMM aged 17–24 is scarce as a prevention method. Moreover, despite the consistent finding that offering HIVST increases HIV testing rates, barriers remain that need to be reduced in order to maximize the potential of this biomedical technology. Such information is necessary to direct implementation efforts to increase HIVST among YSMM, including HIV counseling and linkage to care. The current study was therefore intended to investigate perspectives for HIVST among YSMM and how HIVST can be marketed to increase implementation.

**Methods:**

Between March and September 2020, we enrolled 41 YSMM to participate in one of nine online synchronous focus group discussions about their general experience with HIV preventive services. Guided by the Consolidated Framework (CFIR) for Implementation Research, we explored YSMM perspectives on facilitators and barriers to HIVST implementation. Data were analyzed using a deductive thematic content analysis approach.

**Results:**

Many participants had never used HIVST before their participation in this study (n = 30; 73.2%). Qualitative results exhibited a variety of implementation determinants across the five CFIR 2.0 domains. Barriers included concerns about the format in which the testing materials would be provided (i.e., nature of packaging) and about the method in which the sample would need to be collected, particularly for those who had the testing kit mailed to their home address. These reservations were nested in the fear of unwanted disclosure of their sexual behavior, namely among the respondents who had to cohabitate with family due to the COVID-19 pandemic. Participants also discussed the limited local resources for HIVST. Many participants suggested programs that could be implemented to support HIVST, such as collaborations with trusted community agencies.

**Conclusions:**

Understanding YSMM’ perspectives of HIVST may help identify implementation deficiencies within the delivery system and aid the development of implementation strategies to promote reach of HIVST.

**Table:** 

Text box 1. Contributions to the literature
• Because of the study’s focus to inform strategies for HIVST, understanding implementation determinants encountered by YSMM could improve reach of HIVST.
• As a plausible and novel implementation solution, YSMM in our study discussed ways in which test kit access and distribution could be improved.
• The recommendations provided in our study are especially relevant to reduce some of the permanent challenges resulting from the COVID-19 pandemic, from the way people engage with health care to how services are provided.

## Introduction

Young sexual minority men (YSMM) are disproportionally affected by HIV in the U.S. Of particular concern are YSMM, ages 14–24, who in 2016 made up 83% of all new HIV infections among men [1, 2]. In general, YSMM face unique challenges accessing routine sexual health care due to individual and structural factors, such as limited access to sexual health and gender-affirming care, fear of testing, test-associated and other stigma, and high levels of medical mistrust [3–5]. HIV self-testing (HIVST), whereby a person collects their own sample (oral fluid or blood), performs a rapid test, and then interprets their own result, have the potential to increase uptake and frequency of testing among YSMM [6]. Moreover, HIVST has the potential to circumvent barriers related to visiting health facilities, safeguard confidentiality, and make HIV service delivery systems more responsive to YSMM [7–9].

Though there has been longstanding interest in HIVST, implementation is scarce and poorly documented [10]. Much of the evidence has focused on performance (e.g., sensitivity and specificity), preferences for HIVST methods (e.g., oral fluid vs. fingerstick blood test), acceptability, and willingness. HIVST performance may differ depending on whether the type of specimen collected is oral fluid or fingerstick blood, as well as and whether sample collection is assisted or unassisted by a healthcare provider [7–11]. Though SMM are more likely to perceive blood specimens as more reliable in detecting HIV than oral fluid, SMM most commonly prefer oral fluid methods because it avoids the need to perform fingerstick blood collection with a lancet [7]. However, acceptability for HIVST is less studied among SMM under the age of 24. Data from other key populations suggest that there are a variety of approaches to implementing HIVST that differ based on level of support, level of access, and venues for distribution [12]. Additionally, the 2021 National HIV Behavioral Surveillance System, which used venue-based sampling methods to collect data related to HIV testing in 23 urban areas in the contiguous U.S. and Puerto Rico, suggests that uptake of HIVST was low and limited to roughly 1 in 13 SMM vulnerable to HIV, and rates of HIVST were higher among SMM ages 25–35, more educated, and SMM who had disclosed their sexual identity to a provider [13]. However, it is unclear how YSMM have responded to the use of HIVST. Such information is necessary to direct implementation efforts aimed at increasing HIV testing among this priority population; improving access to HIV testing is one of the four pillars of the U.S. Federal “Ending the HIV Epidemic” Initiative [14].

In recent years, the focus of HIV prevention and treatment research has shifted toward the development of efficient and sustainable program implementation in response to federal priorities [14]. However, most implementation studies for HIVST among YSMM do not involve participants’ perspectives [15]. There remains limited investigation as to why YSMM do not use HIVST or are unable to be reached by current implementation efforts in the U.S. Understanding the perspectives of YSMM in the U.S. may help identify implementation deficiencies within the delivery system, aid in developing or refining strategies to promote dissemination of HIVST technologies and explaining current implementation outcomes. One of the most comprehensive and widely used frameworks to understand implementation outcomes is the Consolidated Framework for Implementation Research (CFIR) [16, 17]. As a determinant (i.e., barriers and facilitators) framework, CFIR specifies 5 constructs that may influence the outcome of implementation efforts: intervention characteristics, outer setting, inner setting, characteristics of individuals, and process [16]. Intervention—or now more commonly referred to as the innovation—characteristics include aspects of HIVST that may affect uptake and persistence; for example, preferences for HIVST methods (e.g., oral fluid vs. fingerstick blood test) may serve as an intervention barrier. Outer setting refers to external influences on HIVST implementation including policy and restricted clinical guidelines. Inner setting includes the characteristics of the implementing clinic, such as the programs or clinicians available to support HIVST uptake. Individual characteristics include individual’s beliefs, knowledge, and other personal attributes that affect HIVST implementation. Finally, process of implementation refers to the planning, execution, and evaluation of HIVST implementation.

Application of the CFIR to the investigation of HIVST implementation determinants among YSMM would not only ensure that no barriers are missed but offer the possibility to compare findings across different settings [18]. Nonetheless, research and evaluation of HIVST with CFIR to date has largely focused on key stakeholders’ perceptions of the implementation and scale-up of HIVST across different international settings, such as in Rwanda [19], Zimbabwe [20], and South Africa [21]. To our knowledge, CFIR has not been used to delineate determinants that influence the outcome of HIVST implementation efforts. This study therefore sought to explore the perspectives of YSMM concerning the implementation and scale-up of HIVST and survey implementation strategies to improve reach of HIVST.

## Method

As described previously [22, 23], participants were recruited online from social media and men-for-men geosocial networking apps between March and September 2020 to participate in one of nine online synchronous focus group discussions (FGDs), which focused specifically on YSMM experiences and attitudes to HIV testing and prevention. Ethical approval was obtained from the Medical College of Wisconsin Institutional Review Board (IRB). A waiver of guardian permission was obtained for those considered minors.

### Participants and procedures

To be eligible to participate in an online FGD, participants were required to: [1] be 17–24 years old; [2] identify as male (including transgender men); [3] report one or more male sexual partners in the past 6 months, including those who identified as transgender; [4] self-report HIV-negative or unknown status; [5] report sexual behavior meeting CDC guideline criteria for increased HIV risk [1], which included the past-6 month behavior of recent bacterial sexually transmitted infection, condomless anal sex (CAS) with a casual male partner, CAS with an HIV-positive or unknown status main partner, or CAS with an HIV-negative main partner who reports CAS with other male partners; and reside in the US. Our age eligibility criteria including YSMM 24 years of age and younger because SMM under age 25 account for the majority of HIV incidence in the U.S. [1], and we only included those age 17 years and older because HIVST is not currently approved by the U.S. Food and Drug Administration for use among individuals 16 years of age and younger [3].

Fraudulent responses were minimized by excluding any information on eligibility criteria from study advertisements and referral mechanisms, using the “prevent multiple ballot box stuffing” feature in Qualtrics to prevent multiple responses, offering no incentive for completion of the screening survey, and using a delayed invitation procedure for the parent study to avoid attempts at determining the study’s eligibility criteria [24]. To further ensure data integrity, duplicates were checked using a procedure of comparing contact information (i.e., name, email, phone number) and IP address.

As described previously [22, 23], individuals who screened eligible received an email invitation to participate. Agreement-to-participate was obtained through a guided procedure using Qualtrics that described the study’s purpose, procedures, and other critical components. Participants then completed a brief quiz as a capacity-to-consent procedure to ensure adequate comprehension of the critical components of consent, including the voluntary nature of the study, risks and benefits to participation, and confidentiality of all data collected. Participants then agreed to participate online, and a copy of the study’s informational letter was emailed to the address of their choosing. Participants were then scheduled for upcoming online group chats, with 6–12 individuals invited per group. FGDs were about 90 min in duration, and participants were compensated with a $40 e-gift card. All chat transcripts were saved for analysis. Despite scheduling 6–12 participants per FG, the actual range of participants per FG was 3–7. While FGs are recommended to have 6–8 participants per discussion [25], there are also known constraints to FGs for marginalized populations, such that some populations (e.g., Latino men) commonly have lower turn out for FGs [26]. Ultimately, all nine FGs provided rich data and were, thus, retained for analysis.

### Focus group content

Participants were asked to report their age, gender identity, sexual orientation, race/ethnicity, and postal ZIP coded into U.S. census region. Participants were also identified by recruitment source. Moreover, a semi-structured focus group guide was used to understand barriers and facilitators to increase access to HIV testing in the context of the COVID-19 pandemic. Content areas for the present study included [1] recent experiences with HIVST [2] attitudes about and experiences with oral fluid- or blood-based HIVST, and [3] adapted public health care initiatives for HIV testing. Example semi-structured interview guide questions applicable to this analysis included: “How do you think people will react to HIV self-testing?”, “What are some of the reasons people might choose to self-test? What are the reasons people might not self-test?”, and “What kind of information might help people self-test with this type of kit and what are some ways we could help support self-testing?”.

### Data analysis

Descriptive statistics were used to characterize the sample using screening survey data. Transcripts were initially coded using MAXQDA, a computer assisted qualitative data analysis software [27]. Before coding, the study team read the transcripts to familiarize themselves with the concepts portrayed by respondents. Codes were identified by the first author, trained in qualitative methods, using a using a combination of deductive and inductive coding to categorize the data [28]. During the initial coding phase, the first and senior author reviewed the audio recordings, and transcripts for salient categories of information until saturation was reached using constant comparative methods [29]. This process allowed us to rate the information saturation in the main topics of the interview [30]. Codes were created by noting overlapping themes in the transcripts and developing code definitions that represented the data. Each transcript was coded and reviewed separately to ensure adequate application of codes. We used a qualitative construct analysis approach from Damschroder and Lowery [31] to rate CFIR constructs related to implementation outcomes. During the initial analytic phase, each analyst separately coded the same randomized transcript with the final codebook and inconsistencies were discussed until agreement was reached. Interrater reliability results revealed a strong agreement with code usage (Κ = 0.82). Coded chat transcripts were then analyzed using thematic content analysis [32] to highlight patterns and identify meaning of the data by the first and senior author. Patterns in themes, including consistent repetition and limited new topics, ensured saturation was achieved. Finally, themes were defined based on CFIR levels and clustering of code application.

## Results

Recruitment activities identified 133 YSMM eligible for our online focus groups. Of the 118 who provided contact information and were invited to participate, 55 consented and 41 participated in an online FGD. Nine FGDs were conducted spanning April–September 2020, with 3–7 participants in each group. Focus group participants were predominantly (85.4%) cisgender men and self-identified as gay (65.9%) or bisexual (29.3%). Average age of participants was 21.0 years, with 36.6% of the sample being under 21 years of age and five participants (12.2%) who were 17 years old. The sample was 26.8% Black and 29.3% Latino, and about half (53.7%) reported their relationship status as single. Most participants had never used HIVST before their participation in this study (n = 30; 73.2%). Eleven participants used HIVST during the COVID-19 pandemic. Full sample characteristics are provided in Table 1.

**Table 1:** Demographics characteristics of young sexual minority men (n = 41)

Continuous Variables	*M*	*SD*
Age (range: 17–24)	21.0	2.5
**Categorical Variables**	***n***	***%***
Gender identity		
Cisgender man	35	85.4
Transgender man	6	14.6
Race/ethnicity		
Black, non-Hispanic	11	26.8
Latino or Hispanic	12	29.3
White, non-Hispanic	14	34.2
Multiracial/another	4	9.8
Sexual orientation		
Gay	27	65.9
Bisexual	12	29.3
Queer	2	4.9
Relationship status		
Single	22	53.7
Partnered	19	46.3
Pre-exposure prophylaxis (PrEP) use status		
Never	25	61.0
Prior PrEP use	7	17.1
Current PrEP use	9	22.0
HIVST use status		
Never	11	26.8
Prior HIVST use	30	73.2
Region		
Midwest	12	29.3
Northeast	11	26.8
South	12	29.3
West	6	14.6
Recruitment source		
Social media	32	78.1
Men-for-men geosocial networking apps	9	22.0

*Note: Percentages may not add up to 100 due to rounding.*

YSMM described barriers to and facilitators of HIVST and adoption related constructs in the innovation characteristics, outer setting, inner setting, characteristics of individuals, and process CFIR domains. Because the study’s primary focus is barriers to HIVST, identified barriers are presented here while facilitators are discussed as strategies to support HIVST. Only relevant CFIR domains and constructs were coded and presented here.

### Innovation characteristics

HIVST itself has attributes that both facilitate and inhibit implementation. A key factor for the innovation is its *relative advantage* over existing traditional clinic-based testing. YSMM users mentioned the favorable privacy and efficiency of using HIVST compared to in person testing. Many shared that they were able to employ HIVST in a way that catered to their own schedules, pandemic restrictions, and testing needs. This capacity did not replace interactions with their primary care provider or other clinic-based testing, but instead supplemented it, allowing some YSMM to test while their clinic was closed and/or had reduced capacity. As noted by one participant:

*I like the option of a quick result. When the lab was backed up at my university because of COVID, sometimes it would take 5 + days for some results. I can see people self-testing, so they can stay at home due to COVID or even medical professionals not trusting testing centers.* (Riley, 21 year, Black)

Barriers included feeling some concern about inaccuracy or conflicting preferences for their preferred and *effective* method for HIVST, as blood or oral fluid-based. Consistent with previous research, blood-based sampling was believed to be more accurate than oral fluid-based (*Evidence Base*). This perception was illustrated in discussions about the differences in window periods between blood-based and oral fluid-based testing. As we discuss later, this sense of timeliness was not discussed as a barrier by all participants but for some it helped inform their preference to use a method that had a shorter window period. As one individual stated:

*The oral test isn’t good for almost 60 days, right? I personally wouldn’t want to take anything less than a month on such a huge test, so I prefer the other method.* (Bill, 21 year, Non-Hispanic, White)

Other discussions around test characteristics included the technique and/or sample collection method (*Design*). Specifically, many YSMM discussed their distaste for having to prick their own finger (e.g., *for me I am a bit high maintenance. Not only do you have to bleed all over the card, but I do it all? No thanks*). Further, in their discussions of using a needle and/or lancet, some YSMM were concerned about how much blood they would need to collect and/or whether it mattered which finger was used to collect their sample (*Complexity).* As one participant plainly said:

*I have never used a finger prick anything before, so I would definitely be nervous about the pain. Plus, I’m left-handed but use my right hand for some things, so I wouldn’t know which finger to prick and would spend way too long deliberating.* (Camilo, 20 year, Latino)

When considering oral fluid-based HIVST, some participants had serious concerns about the packaging of the box (*Design*). For instance, one participant noted that when he went to purchase the *OraQuick* HIVST kit, the cashier asked him invasive questions when he saw for what the test was used. For other respondents, their concern was like those reported with blood-based HIVST; in that, their apprehension was fear that the packaging would *out* them to their parents by association. Indeed, many YSMM said that they would not know how to explain the test to their parents or would be worried about going to a clinic to get the test kit. As two participants put it:

*I feel like some people might feel insecure about picking the test up from a community organization, as your presence might imply your association with it, especially when you are in a small town.* (Joe, 20 year, Non-Hispanic White)

*Discreet packaging is key. I was worried about my parents opening my mail when I got my OraQuick. Also, sometimes the tests are just broken or defected on arrival and give confusing results.* (Tim, 24 year, Non-Hispanic White)

### Outer setting

Challenges related to the outer setting of the innovation, such as the COVID-19 pandemic (*Critical Incidents)*, preferring support from friends and community members rather than medical providers, and medical mistrust (*Local Attitudes)* arose as well. Participants’ willingness to use HIVST varied substantially due to the various practical and technical challenges brought forth by the COVID-19 pandemic. Especially among YSMM who were not out to their families about their sexual orientation, privacy and fear was constructed as a barrier to HIVST, including how to mail their sample and where within the house to conduct the HIV test. To some, limited privacy meant no longer having a secure place to have a test kit mailed to their home address or having to ship the testing materials to a more secure location. As one participant described:

*The mailing system is vulnerable beyond just your roommate or family member opening your mail. Some people may not have a secure location for their testing kit to arrive to. Postmen just drop things on your city doorstep in cities I have lived in.* (Tim, 24 year, Non-Hispanic White)

Similarly, in a different FG, there was apprehension about how the kits would be mailed to their home:

*I would have reservations of mailing the kit to me since moving back home. I would rather pick it up in the city while getting PrEP.* (Joe, 19 year, Latino)

*I would hate to accidentally come out to my family from mail. My family definitely goes through my mail.* (Dante, 24 year, Multiracial)

*If the test were mailed, I would hope to see it in packaging that wouldn’t alert my parents.* (Robert, 22 year, Latino)

Moreover, despite these barriers from the COVID-19 pandemic, there were also several practical benefits to HIVST as an alternative method to testing given the restricted COVID-19 guidelines and policies regarding in-person testing (*Policies & Laws)*. YSMM who were displaced because of the pandemic commonly described HIVST as convenient in the form of location of testing, greater control over their health, and efficiency. Some youth were even observed within FGs to recommend HIVST to other study participants given their positive experience with the test during the COVID-19 pandemic (*Partnerships & Connections*). Finally, for the outer setting, which encompasses the broader environment and policy, YSMM brought up lack of inclusion in clinical research, advertisements, and the missed opportunity of not embedding HIVST education into STI/HIV prevention interventions.

### Inner setting

The inner setting for HIVST implementation is the specific clinical settings that currently treat and/or provide HIVST. One overarching inner setting barrier, seen across all FGDs, is feeling that providers are unable to accommodate YSMM social and cultural needs (*Recipient-Centeredness* and *Equality-Centeredness*), feeling that primary care appointments are challenging to schedule or too short to address social or mental health concerns and not receiving adequate or accurate information about HIVST (e.g., participant thought HIVST was ineffective and not recommended) (*Available Resources*). For instance, many YSMM attributed these concerns to more specific questions of how an oral fluid sample would have the capability to test for HIV, as they considered HIV to be transmitted through blood. Besides concern over the effectiveness of an oral fluid sample, some participants also described the mechanism the test uses to check for antibodies. One participant noted the following as his concern for oral-based HIVST:

*So, it’s a saliva test which is notorious for not being useful in the heat of the moment. It’s basically testing your body’s reaction to the virus in saliva. I feel like the way they’re designed is inefficient for the job they need to do. I would never take the saliva test over just going to my doctor and having my blood taken.* (Richard, 24 year, Black).

### Characteristics of individuals

YSMM reported several perceived patient-level barriers across uptake with HIVST that are categorized as individual characteristics. Affecting HIVST awareness and uptake, participants commonly highlighted the reduced privacy they had at home and fear, especially among YSMM who were not out to their families about their sexual orientation (*Innovation Recipient).* While in the minority, some YSMM described heightened fears and/or stress when they discussed the time needed for their sample to be picked up, delivered to the clinic and then the resultant process for their sample to be processed. Moreover, there was also concern for potential harm and cross-contamination that were compounded by the COVID-19 pandemic. For these YSMM, they expressed concern for the cleanliness and complications in collecting their own blood with a card without the support of a clinician, which reduced their *motivation* to use HIVST. As some noted:

*If I am having something pretty invasive being done, I’d much rather just have a medical professional supervise so then I could get any questions answered immediately too and make sure that it is being done correctly.* (Kalen, 20 year, Black)

*I’d be worried about contamination, especially since it’s on this card.* (Kenny, 23 year, Multiracial)

*I think people might feel a little weird covering something of theirs with blood. Considering it is a method of transmission for so many things.* (Steven, 24 year, Non-Hispanic White)

Nonetheless, YSMM also expressed the option to use HIVST on their own on an ongoing basis, as opposed to ongoing, clinician-delivered testing, requiring appointments with your health care provider, and at times, a different appointment for lab testing. Similarly, YSMM appreciated the practical benefits, describing it as a method to avoid potentially awkward or challenging conversations with providers (e.g., “*the privacy it provides made me feel more comfortable getting tested, since I didn’t need to have face-to-face communication.”*).

Although some participants seemed unwilling to consider oral fluid-based HIVST, others reflected on the positive practical, psychological, and social benefits associated with HIVST (*Self-Efficacy, Knowledge, Attitudes, and Beliefs*). For example, the primary perceived benefit for oral fluid-based HIVST was that it was assumed to be more useful for YSMM who were concerned about privacy and confidentiality when accessing testing at a clinic or hospital. Oral fluid testing was widely assumed to be more accessible than other forms of HIVST to YSMM who otherwise found it challenging to coordinate testing (e.g., *I prefer the comfort of knowing not only quickly but also without judgment and in privacy*). As previously noted, blood-based sampling was believed to be more accurate than oral fluid testing, but many respondents preferred the method that was painless and less invasive. A final point to be made regarding the motivation to use oral-based HIVST relates to the speed with which a test could be done, and result obtained. The opportunity to test, when they had time (e.g., *I don’t have to worry about calling out of work or class to get tested or going in before 5p*) and wherever they were, was highly valued. Indeed, some YSMM also noted the benefits of testing in the *moment* with your partner. These benefits are illustrated below:

*I’ve used it and I find it’s simple, no waiting around an office, no waiting on online results to be updated.* (Don, 21 year, Non-Hispanic White)

*I like it because finding time in my schedule to go to a clinic (which usually are only open when I’m working) is hard, and because it takes a lot of time to drive there, wait for an hour in the lobby because they’re always slow, and then get seen.* (Jason, 22 year, Latino)

*I like the option of a quick result in the heat of the moment for some comfort; I am a super big proponent of testing with partners.* (Jay, 24 year, Non-Hispanic White)

*I prefer the oral swab. Imagine being able to have one with you at home to use if you want a result before hooking up with someone new (obviously knowing about the 3mo window).* (Parker, 23 year, Non-Hispanic White).

### Process

Process barriers were less discussed, some barriers included a delay in not receiving a follow-up call after requesting for additional information for HIVST and receiving a damaged testing kit.

### Implementation recommendations to support HIVST

The final theme entailed broader implementation strategies or facilitators to support HIVST. Some points that were highlighted as important to communicate in future messaging to support HIVST were: (1) how their blood sample is mailed/protected; (2) the time required for the test to be mailed, delivered, and interpreted; (3) who will have access to their sample; and [4] how their sample will be stored/destroyed. One participant shared:

*People may want to know where their biological material is stored and for how long, who will have access to it. Will they be able to track its “progression” in the medical pipeline? That would be slick. Like tracking a package but it’s your biomaterial from your mailbox to its later incineration or whatever.* (Miguel, 20 year, Latino)

The majority of the participants also expressed concern for how their results would be communicated for blood-based HIVST. YSMM proposed that they be given the option to decide their preferred method of communication for their results (Tailoring). Some of these options included through a protected e-mail, text message, voice message, virtual portal, and/or with a representative on the phone or in person. They advised that these methods would be sensitive to potential concerns for privacy and accessibility. As some respondents explained regarding their anticipated HIVST in the future:

*I would want the results by email preferably, to maintain privacy and accessibility to the results. You don’t know who will get to the mail that day, so I would prefer to have control.* (Paul, 22, Non-Hispanic White)

*I always worry about missing a call about my results, because I never pick up my phone to unknown numbers. I would definitely want to receive my results in writing via text or email.* (John, 23 year Non-Hispanic White)

*I would be okay with that, but I can imagine some people being afraid of that text message outing them. Maybe a text message saying check your email for test results or something else vague.* (Carlos, 19 year, Black)

Virtual communication was discussed as an additional method to increase education through infographics that would be shared by e-mail and/or that could be accessed online. For one participant, such a method could be used to share information about the test with their social support or to inform their sexual partners (e.g., *information and strategies about bringing this up this test with current or future sexual partners. Maybe even a script*).

Beyond recommendations for packaging and delivery, many participants also described strategies to increase motivation, acceptance, and knowledge about oral fluid-based HIVST. Strategies to increase motivation and acceptance were largely specific to visual techniques to increase awareness and familiarity of how effective oral fluid-based methods are for HIV testing. Building on the concern for how minimal information is advertised for HIVST (e.g., I have never seen advertisements for self-testing units), participants in a different FG discussed strategies for implementation with visual aid:

*I think adding a cool band aid or a motivational card could help people be more down for this as well. I know it seems silly, but Avocado band aids are how I get through my shot days more recently.* (Brian, 23 year, Non-Hispanic White)

*When I did a finger prick test at home, the pictures were super helpful. I love the idea for stickers, cool information, and pictures.* (Michael, 21 year, Non-Hispanic white).

Complementing the perceived acceptance and awareness of oral fluid-based HIVST, some participants also described that visual strategies would support their confidence in HIVST administration. While many participants seemed to understand the directions for how to collect their sample and the education about the ‘window period’ during which the test’s sensitivity is low, some had concerns about the reliability of HIVST and improper sample collection. Many recommended *testimonials* or videos of *real* people using the test to provide factual information and potentially open, group conversations to discuss questions and/or fears (Engaging).

*I wouldn’t want pages upon pages of written text. Too overwhelming. I wouldn’t want excessive medical talk. Explain it in simple terms for those that are well not equipped with medical knowledge and use real people.* (Conner, 20 year, Non-Hispanic White)

*I think an interactive component could keep people engaged over multiple blocks and feel connected.* (Joe, 23 year, Black)

## Discussion

The present study examined YSMM preferences, as well as factors likely to influence uptake of HIVST. We found practical benefits associated with HIVST and some preferring HIVST to traditional clinic-based testing. Because of the study’s focus to inform strategies for HIVST, understanding implementation determinants encountered by YSMM could improve reach of HIVST. Findings for the current study can therefore be considered using the determinants of the CFIR [16]. Applying the CFIR framework can help identify where key changes can be made and highlight strategies that are effective to improve HIVST implementation for YSMM (Table 2). As such, we discuss our findings using CFIR to better organize determinants and areas for new or expanded implementation strategies.

**Table 2:** Young Sexual Minority Men Sample Quotes by the Consolidated Framework for Implementation Research

CFIR Domain	Sample Quote
**Intervention Characteristics**: Aspects of HIVST that may affect uptake and persistence, for example method of testing (e.g., oral fluid vs. fingerstick blood test)	*“I do not like getting poked with needles or anything similar so I do not think I would be able to conduct that test on my own. I am squeamish and I would need someone else to conduct that test on me”*
**Outer Setting** External influences on HIVST implementation including economic, environmental, or political conditions that enable the Outer Setting to impact implementation or delivery of the innovation	*“When I used the home blood test it was a huge problem because when I mailed it back to the lab to get results the lab closed and wasn’t able to receive the testing results…The very next day after sending all of the samples in they sent an email asking me to not send it in due to the pandemic”*
**Inner Setting** Characteristics of the implementing clinic, such as programs or resources addressing the needs and welfare of recipients	*“I wouldn’t want pages upon pages of written text. Too overwhelming. I wouldn’t want excessive medical talk. Explain it in simple terms for those that are well not equipped with medical knowledge and use real people”*
**Characteristics of Individuals** Individual beliefs, knowledge, and other personal attributes that affect HIVST implementation	*“I would want the results by email preferably, to maintain privacy and accessibility to the results. You don’t know who will get to the mail that day, so I would prefer to have control”*
**Process** The planning, execution, and delivery of HIVST implementation	*“I feel like some people might feel insecure about picking the test up from a community organization, as your presence might imply your association with it, especially when you are in a small town”*

Barriers and facilitators related to a number of CFIR Innovation Characteristics, Outer Setting, Inner Setting, Characteristics of Individuals, and Process domains and constructs [16]. Across FGDs, many participants expressed significant concerns for the method of testing (CFIR Domain: Innovation Characteristics) that affect uptake and persistence among YSMM. Barriers included concerns about the format in which the testing materials would be provided (i.e., nature of packaging) and about the method in which the sample would need to be collected, particularly for those who had the testing kit mailed to their home address or for those who would have to send their blood sample in the mail. These reservations were nested in the fear of unwanted disclosure of their sexual behavior, namely among the respondents who had to cohabitate with family due to the COVID-19 pandemic (CFIR Domain: Outer Setting). In comparison, participants in previous studies prior to the pandemic (e.g., 2017) found participants to be more likely to uptake HIVST due to perceptions of increased privacy [33]. Participants also discussed the limited local resources for HIVST (CFIR Domain: Inner Setting). Many participants suggested programs that could be implemented to support HIVST, such as collaborations with trusted community agencies. Related to method of HIVST, participants throughout each FGD expressed concerns about the validity of the oral sample, believing that using a blood-based test would be more effective (CFIR Domain: Individual Characteristics). Concerns about accuracy of oral testing have been found in previous studies, as well [34]. Similarly, YSMM expressed some concern regarding the limited support and/or counseling available (CFIR Domain: Process), which has previously been identified as a barrier to uptake in samples outside the US [35].

As a plausible and novel implementation solution, YSMM in our study discussed ways in which test kit access and distribution could be improved. For instance, in our study, packaging the kit at a local community center or discreet packaging were suggested as important factors that may promote uptake with HIVST. Some participants suggested repackaging the kit to be more representative of youth experiences, which includes adding relatable graphic designs for youth or promotional materials. Some were interested in a health pass or form of identification like the concept of a “vaccination passport” for COVID-19 that would communicate to others that they were tested. Study participants suggested that such communication strategies would increase uptake of HIVST among young people, because they would be more appealing to young people and could potentially reduce the stigma around HIV testing.

Our results are consistent with reviews that have found higher HIVST uptake when privacy and confidentiality are prioritized in kit distribution [8–10, 36]. In addition, participants were opposed to having detailed or lengthy texts in the instruction manual, as some respondents expressed fatigue of health information from COVID-19 [33, 34]. When considering information and/or education for HIVST, the method of presentation was suggested as an important factor that may increase familiarity and comfort with testing. A few participants noted that virtual technologies could be one useful mechanism to communicate the effectiveness of each method, while also being more relatable to youth. This is consistent with a recent study that found app-based messaging and virtual education for YSMM in HIVST to be preferred over traditional material [37]. Little is known about how the combination of these implementation strategies proposed by YSMM would ultimately affect HIVST outcomes among YSMM. Future studies are therefore needed to evaluate implementation strategies on the use of HIVST.

Taken together, our findings indicate that HIVST may have the potential to increase HIV testing among youth with less access to traditional testing. The recommendations provided in our study are especially relevant to reduce some of the permanent challenges resulting from the COVID-19 pandemic, from the way people engage with health care to how services are provided. Moreover, prior reports documented disruptions to biomedical HIV prevention during the pandemic [2, 38, 39]. However, many findings from this study will be relevant outside of the constraints imposed by the COVID-19 pandemic. As recorded elsewhere [40], there has been an immense increase in telehealth/virtual care since the beginning of the COVID-19 pandemic. One potential strategy to help support HIVST in this post-pandemic setting is to provide consultations and support for HIVST through telecommunication technologies. For most people, virtual care is accessible, simple to use, and reliable [40]. Telecommunication for HIVST has the potential to decrease fear among potential testers who are worried about limited support because support can be offered in real time. Additional technologies could also consider an anonymous platform for testers to share their experiences and concerns with HIVST to further foster community-level support.

Our study has implications for current research and public health implementation programs. From a research perspective, our findings suggest the need for more extensive research on HIVST for youth who reside with their parents or have greater privacy concerns. From a public health program perspective, additional methods are needed for YSMM to pick up testing materials and a safer place to send their sample collection. Given how many YSMM have been displaced because of the COVID-19 pandemic, one method to dispense HIVST materials may be through public vending machines [41–43]. From the perspective of HIVST programs, our data may be useful for optimizing pilot vending machine HIVST sites, marketing strategies, and service delivery models. When asked specifically about HIVST, some patient characteristics (age, housing security, outness) were viewed as an implementation barrier. HIVST implementors could, for example, provide confidential phone consultations to YSMM who are unsure how to collect their own sample. The implications of our findings for scale-up are even clearer when placed within the context of implementation strategies identified by the Expert Recommendations for Implementing Change (ERIC) study [44]. For example, ‘*intervene with patients/consumers to enhance uptake and adherence’* and ‘*use mass media*’ is akin to our finding for enhanced educational methods through infographics. Building on the barrier for how minimal information is advertised for HIVST, user feedback will be used to tailor the implementation strategy to the unique needs of HIVST for YSMM.

Our results should be interpreted with some caution. First, we recruited a convenience sample online, which may have been subject to selection bias, potentially limiting the generalizability of study findings. Other limitations were inherent to the FG methodology, such as size and the challenge of keeping FGs focused on the discussion. Albeit unsuccessful, attempts were made to keep FGs with a similar number of participant(s) in each group, we had some participant(s) drop out or not login on time. Second, we had a limited number of participants who identified as gender minorities; as such, there is a need for further research to fully understand the challenges of HIVST for this population. Third, FGDs were conducted using a chat-based format, which could have limited FGD facilitators’ ability to use non-verbal cues to guide additional probing. However, we believe this effect to be minimal regarding the identification and richness of themes when compared to in-person FGDs [45]. Finally, qualitative findings are highly subjective [46, 47]. Nonetheless, various researchers were involved throughout the entirety of the project, from FGD facilitation to the coding to ensure credibility and dependability of the study findings. We further enhanced the credibility of our findings by following rigorous thematic content analysis and interpretation of the data that were extensively discussed and validated by the research team.

## Conclusions

The current study builds on emerging evidence about the barriers to HIVST among YSMM. In order to maximize the uptake, response, and effectiveness of HIVST, it is necessary to address the unique COVID-related barriers and viewpoints of YSMM to understand their preferences, needs, and concerns, and to build interventions that are sensitive to these characteristics. Such findings are especially necessary given the public health changes brought forth by the COVID-19 pandemic and will be useful in the post-COVID era. Continued user feedback will be necessary to ensure that prevention efforts are aligned with the growing challenges and changes to HIV prevention and testing.

## Data Availability

Data is not accessible to the public to minimize the risk of loss of confidentiality. Data is available upon request to the Administrative Core of the Health Intervention Sciences Group / Center for AIDS Intervention Research. Individuals who meet criteria for access to de-identified data should contact the Principal Investigator (sjohn@mcw.edu) or Kevin Brown (kdbrown@mcw.edu) to facilitate data transfer.
